# Nanostructure of Unconventional Liquid Crystals Investigated by Synchrotron Radiation

**DOI:** 10.3390/nano10091679

**Published:** 2020-08-26

**Authors:** Francesco Vita, Fabrizio Corrado Adamo, Michela Pisani, Oriano Francescangeli

**Affiliations:** Dipartimento di Scienze e Ingegneria della Materia, dell’Ambiente ed Urbanistica, Università Politecnica delle Marche, via Brecce Bianche, 60131 Ancona, Italy; f.vita@univpm.it (F.V.); f.c.adamo@staff.univpm.it (F.C.A.); m.pisani@univpm.it (M.P.)

**Keywords:** synchrotron X-ray diffraction, soft matter, liquid crystals, bent-core mesogens, cybotactic nematic, all-aromatic liquid crystals, polymer-dispersed liquid crystals

## Abstract

The macroscopic properties of novel liquid crystal (LC) systems—LCs with unconventional molecular structure as well as conventional LCs in unconventional geometries—directly descend from their mesoscopic structural organization. While X-ray diffraction (XRD) is an obvious choice to investigate their nanoscale structure, conventional diffractometry is often hampered by experimental difficulties: the low scattering power and short-range positional order of the materials, resulting in weak and diffuse diffraction features; the need to perform measurements in challenging conditions, e.g., under magnetic and/or electric fields, on thin films, or at high temperatures; and the necessity to probe micron-sized volumes to tell the local structural properties from their macroscopic average. Synchrotron XRD allows these problems to be circumvented thanks to the superior diffraction capabilities (brilliance, *q*-range, energy and space resolution) and advanced sample environment available at synchrotron beamlines. Here, we highlight the potentiality of synchrotron XRD in the field of LCs by reviewing a selection of experiments on three unconventional LC systems: the potentially biaxial and polar nematic phase of bent-core mesogens; the very high-temperature nematic phase of all-aromatic LCs; and polymer-dispersed liquid crystals. In all these cases, synchrotron XRD unveils subtle nanostructural features that are reflected into macroscopic properties of great interest from both fundamental and technological points of view.

## 1. Introduction

Soft matter [1] is central to a number of promising advanced technological applications spanning across physics, chemistry, biology, and medicine. These include ultra-fast displays, novel photonic devices for optical data storage and processing, micro- and nano-fluidic devices, nano-electrochemical systems, active matter, chemical–biological sensing, novel mechanisms for drug delivery, and innovative medical therapies. Among the variety of soft materials currently investigated, liquid crystals (LCs) [2] play a prominent role because of the wealth of supramolecular structures that they exhibit and their potential to reflect specific nanostructural features into unconventional macroscopic properties.

LCs have been studied extensively in the past decades for both their peculiar nanostructure and physical properties. However, these investigations have mainly concerned the most traditional families of compounds, known as calamitic (rod-like) and discotic (disc-like) LCs. In recent years, the ability to synthesize mesogenic molecules with unconventional geometrical shapes and the development of processes to produce composite materials containing LCs have paved the way for the realization of new mesogenic compounds with unique properties. This has revived interest in the investigation of the structure–function relationships in LCs with the aim of finding connections between the nanoscale structure and the macroscopic behavior of these materials. Synchrotron X-ray diffraction (XRD) is the most powerful tool to probe the structure of matter with atomic resolution; therefore, it is extensively used to this purpose.

In this paper, we review a number of recent experimental studies performed to investigate the nanoscale structure of new unconventional LCs and LC-based soft materials, exhibiting unique physical properties of high fundamental and/or technological impact. While X-ray techniques have been applied to the characterization of a broad array of LC systems, here we will focus on thermotropic nematics, the most studied class of LCs, inevitably neglecting a number of other interesting systems such as smectic LCs, lyotropic LCs, LC polymers, chiral LCs, colloidal LCs, etc. Most of the reported experiments have been carried out at the European Synchrotron Radiation Facility (ESRF), Grenoble, France. In all cases, synchrotron radiation combined with an ad hoc experimental setup has made it possible to investigate the details of the mesophase inherent LC order (orientational and positional) responsible for these peculiar properties.

The first section concerns new LCs belonging to the class of bent-core (or banana-shaped) mesogens, which are of great potential for the realization of the elusive and long sought-after ferroelectric and biaxial nematic phases. The second section reports recent advances in the study of lath-like all-aromatic mesogens that represent a prototypical embodiment of the ideal rigid rod mesogen, i.e., the basic ingredient of most of the theories of the nematic (N) phase. The third section concerns the study of confined N order in the class of polymer–matrix composite materials known as polymer-dispersed liquid crystals (PDLCs). In all cases, the unconventional properties of the investigated materials result from the peculiar orientational and/or positional order of the N mesophases specifically involved.

## 2. Bent-Core Mesogens 

### 2.1. Materials and Methods

**Scheme 1:**
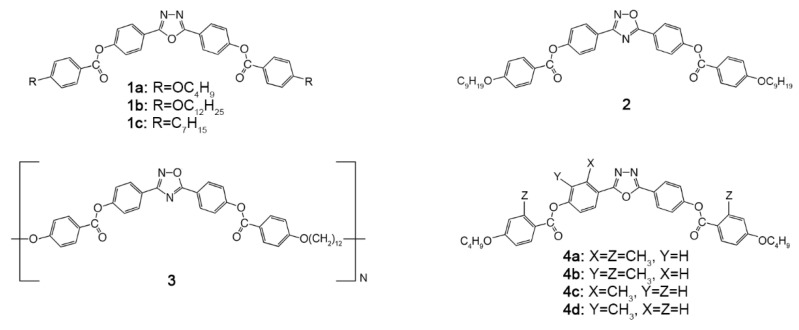
Chemical structures of investigated bent-core mesogens (BCMs).

Bent-core mesogens (BCMs) are bent-shaped molecules typically consisting of a bent aromatic core linked to two alkyl terminal chains (Scheme 1) [3,4]. They form thermotropic LC phases exhibiting physical properties substantially different from those of traditional linear (calamitic) mesogens. The N phase in BCMs was discovered in 2000 [5], and it has soon become the most promising candidate in the quest for the elusive polar ferroelectric nematic (N_f_) [6] and biaxial nematic (N_b_) phases [7,8].

**Figure 1:**
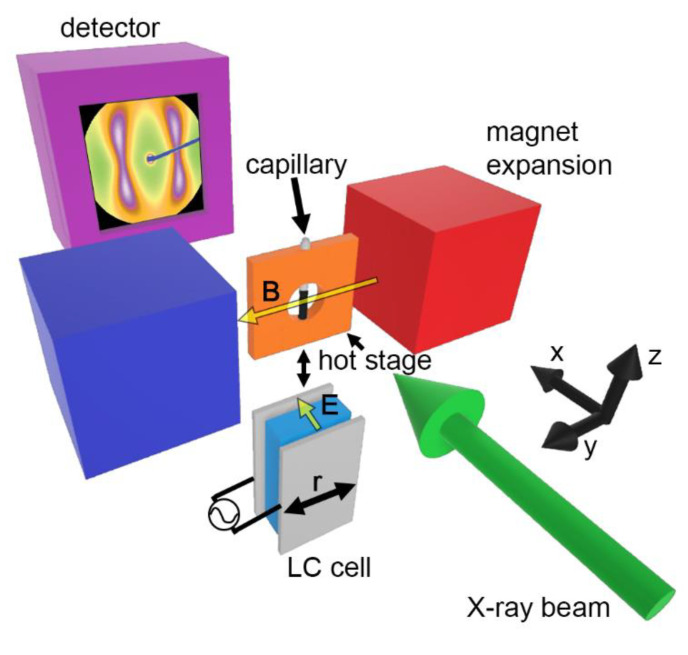
Experimental setup for XRD measurements on BCMs (capillary and cell samples) under a maximum magnetic field of 1 T. By using a different pair of magnet expansions, it was possible to have the magnetic field parallel to the X-ray beam. Alternatively, a superconducting magnet allowed a magnetic field up to 4 T to be applied orthogonal to the beam in the vertical direction. The hot stage allowed measurements on both capillaries and liquid crystal (LC) cells. In the latter case, an electric field could be applied across the cell, parallel to the incident X-ray beam.

Synchrotron radiation has been used since 2002 [9] to probe the nanoscale structure of the N phase of BCMs such as those reported in Scheme 1. The experimental setup used in most of these experiments and schematically represented in Figure 1 was designed to allow XRD measurements at variable temperature over a relatively broad range of the scattering wave-vector **q**, which is typically comprised between about 1 and 20 nm^−1^. The sample holder allowed the insertion of either a capillary for bulk powder sample measurements or a thin cell for surface-oriented sample measurements. Cells (typical thickness between 10 and 20 µm) were prepared using two ultrathin (100 µm) glass plates coated with a conductive film of indium tin oxide (ITO). These glass plates were further coated with a thin film of SiO_x_ deposited under vacuum at a 60° evaporation angle, in order to achieve strong planar anchoring with homogeneous in-plane orientation of the nematic director **n** parallel to a reference direction **r**. Then, the cells were assembled with the glass plates facing their coated sides in a parallel-plane configuration and separated by high-precision spacers. Finally, the cells (1 cm x 1 cm) were filled by capillarity with the LC in the fluid isotropic phase and then slowly cooled down to room temperature. Capillary and cell samples were mounted on a special temperature-controlled (± 0.1 °C) hot stage, allowing the insertion of a static magnetic field **B** of variable intensity (up to 1 T by using permanent magnets, up to 4 T by means of a superconducting magnet [10]), either perpendicular to the incident X-ray beam, as shown in Figure 1, or parallel to it. In the case of cells, an electric field **E** parallel to the X-ray beam could be applied across the conductive plates. XRD measurements were carried out on samples aligned under the single or combined actions of **B**, **E** and the surface anchoring field.

### 2.2. Cybotactic Order

**Figure 2:**
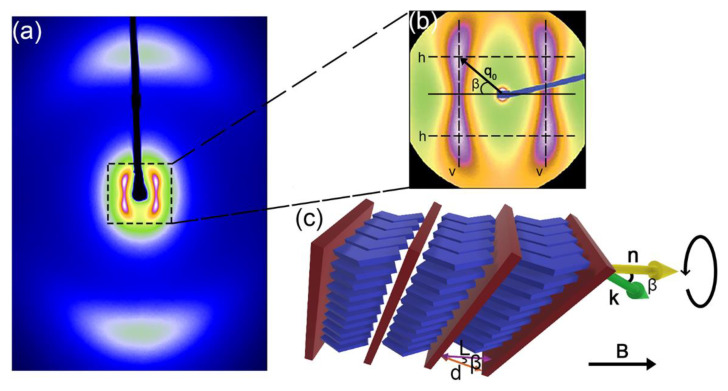
(**a**) Representative wide-angle XRD pattern of BCM skewed cybotactic nematic phase, here measured for compound **1a** aligned by a horizontal magnetic field. (**b**) Low-angle XRD pattern of the same sample, measured at the same conditions of temperature and magnetic field. (**c**) Schematic diagram of a skewed cybotactic group (symbols are defined in the text). The layer normal **k** of each cluster is randomly oriented around the nematic director **n** (aligned by the magnetic field **B**) and forms with it an angle *β*.

As a representative example of the XRD patterns typically observed in the N phase of BCMs, Figure 2a,b show, respectively, the wide-angle and low-angle diffraction patterns from a bulk capillary sample of BCM **1a** (Scheme 1), uniaxially aligned by a horizontal magnetic field **B** (*B* = 1 T) perpendicular to the X-ray beam. The wide-angle pattern (Figure 2a) exhibits a pair of diffuse crescents centered on the equator (the vertical axis orthogonal to **B** in Figure 2a,b), which is typical of the short-range (liquid-like) molecular positional correlations in the direction transverse to the aligned nematic director **n** ‖ **B**. On the other hand, the small-angle reflections (Figure 2b), which in conventional nematics are centered on the meridian (the horizontal axis parallel to **B** in Figure 2a,b), exhibit a peculiar azimuthal splitting into two pairs of diffuse spots (four-spot pattern), which are symmetrically located about the equatorial and the meridional axes. Similar XRD data for BCM **2** were originally interpreted in terms of a skewed cybotactic structure of the N phase, consisting of nano-sized clusters of molecules, the cybotactic groups, each featuring a combination of short-range smectic C (SmC)-like (i.e., layered and tilted) positional order and biaxial, possibly polar, orientational order [11]. Contrary to the initial interpretation of the four-spot pattern in BCMs, assuming a nonlinear molecular form factor together with a biaxial N structure factor [12,13], the cybotactic model accounts for the low-angle splitting in terms of the intrinsic tilted (SmC-like) supramolecular layered structure of the cybotactic clusters [14,15,16].

The schematic picture in Figure 2c connects the structural parameters of a single skewed (i.e., SmC-like) cybotactic cluster with the corresponding XRD parameters. The magnetic field aligns the clusters so that the average direction of the long molecular axis, hence the director **n**, is parallel to **B**. This agrees with the positive diamagnetic anisotropy of most BCMs deduced from nuclear magnetic resonance (NMR) experiments, and it is also confirmed by the equatorial position of the wide-angle reflections discussed above (Figure 2a). Under a magnetic field, the normal to the smectic layers, **k**, is randomly distributed about **B** at the tilt angle *β*. The scattering that results from such distribution of microscopic SmC-like clusters leads to the XRD pattern of Figure 2b, where the low-angle signal is split into two pairs of symmetric diffuse spots. Indicating with **q**_0_ the scattering vector corresponding to the maximum of each spot, the angle between **q**_0_ and the meridional direction corresponds to the molecular tilt angle *β*, while the layer spacing *d* is given by *d* = 2*π*/*q*_0_; *d*/cos *β* provides the molecular length *L*.

**Figure 3:**
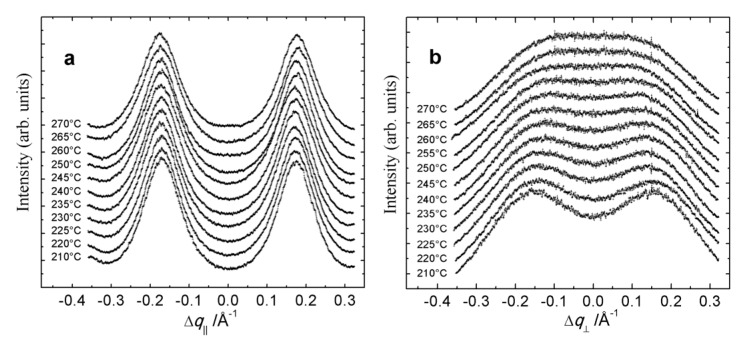
Intensity profiles (**a**) *I*(Δq_‖_) and (**b**) *I*(Δ*q*_⊥_) of the low-angle diffuse spots measured through the maxima at **q_0_** along the longitudinal (‖ **B**) and transverse (⊥ **B**) directions, respectively, at different temperatures in the skewed cybotactic N phase of BCM **1a**. Reproduced from [14] with permission from The Royal Society of Chemistry.

An estimate of the average size of the cybotactic clusters can be obtained from the longitudinal (‖ **B**) and transversal (⊥ **B**) intensity profiles of the four-spot pattern. As an example, Figure 3a,b show the scattered intensity *I* as a function of Δ*q*_‖_= ±∣**q** -**q**_0__⊥_∣ and Δ*q*_⊥_= ±∣**q** -**q**_0__‖_∣ measured for BCM **1a** by performing, respectively, horizontal or vertical scans of the four-spot pattern along the straight *h* and *v* lines shown in Figure 2b [14]. These curves feature a pair of broad symmetric peaks whose full width at half maximum (FWHM), *δq*, is inherently connected to the correlation length *ξ*, namely the quantity that measures the length-scale over which particle–particle positional correlations are lost. Following the procedure outlined in [14], the longitudinal (‖ **B**) and transversal (⊥ **B**) correlation lengths, *ξ*_‖_ and *ξ*_⊥_, characterizing the anisotropic short-range positional order of the cybotactic clusters, were calculated as *ξ*_‖_ = 2/*δq*_‖_ and *ξ*_⊥_ = 2/*δq*_⊥_, respectively. These estimates should be considered as lower limits for the actual correlation lengths. In fact, measured values, especially in the transverse direction, are inevitably underestimated by the broadening of the low-angle reflections caused by orientational disorder [17].

**Figure 4:**
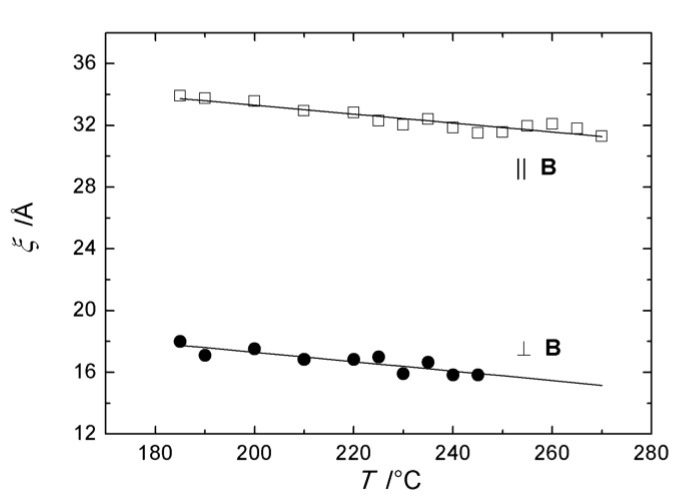
Longitudinal, *ξ*_‖_ (open squares), and transverse, *ξ*_⊥_ (full circles), correlation lengths as a function of the temperature in the cybotactic N phase of BCM **1a**. The solid lines are linear fits to the experimental data. Reproduced from [14] with permission from The Royal Society of Chemistry.

Figure 4 shows the values of *ξ*_‖_ and *ξ*_⊥_ so obtained as a function of temperature: values of *ξ*_‖_ and *ξ*_⊥_ of the order of one molecular length *L* (approximately 36 Å) and a few (roughly three) intermolecular distances *D* (typical intermolecular distance approximately 4.5 Å), respectively, are to be interpreted as indicative of very short-ranged correlations. Based on the quasi-Lorentzian lineshape of the peaks in Figure 3, the longitudinal and transversal cluster sizes, *D*_‖_ and *D*_⊥_, could be estimated as *D*_‖_ = 3*ξ*_‖_ ≈ 3*L* and *D*_⊥_ = 3*ξ*_⊥_ ≈ 10*D* [14]. Very similar values were found for most of the BCMs investigated so far, which represents a direct proof of the nanometer size of cybotactic clusters: estimating their volume as *D*_⊥_ × *D*_⊥_ × *D*_‖_, they thus comprise an average number of molecules of the order of 3 × 10 × 10 ≈ 300, i.e., a few hundred molecules. It must be emphasized that cybotactic groups are present throughout the BCM N temperature range, even when no underlying Sm phase is present. This evidence, in conjunction with the absence of divergence in the correlation lengths on approaching the N–Sm transition, strongly differentiates BCM cybotaxis from the pretransitional cybotaxis that is observed in conventional nematics [16,18].

**Figure 5:**
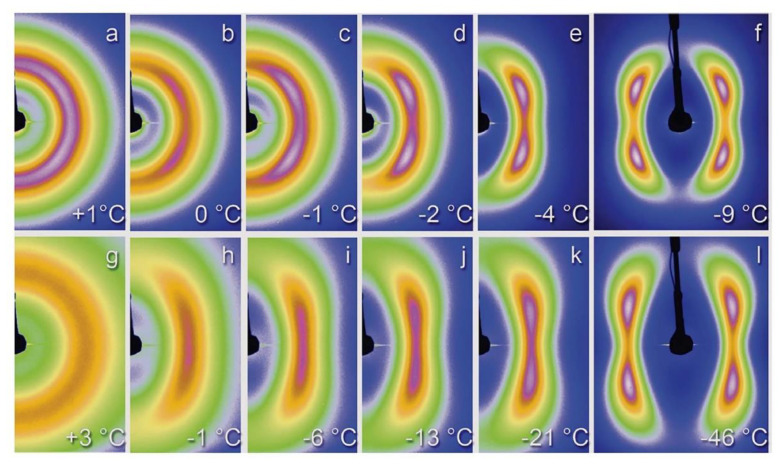
Low-angle XRD patterns of two representative BCMs, acquired on cooling from the isotropic melt under a horizontal magnetic field at different values of the reduced temperature *T* − *T_N–I_*: (**a**–**f**) BCM **1b**; (**g**–**l**) BCM **1c**. Reproduced from [16] with permission from The Royal Society of Chemistry.

Figure 5 shows the temperature evolution of the low-angle XRD patterns of two representative BCMs based on the 1,3,4-oxadiazole bisphenol (ODBP) moiety, namely mesogens **1b** and **1c** in Scheme 1. In Figure 5a–f (BCM **1b**), the four-spot pattern is apparent at all temperatures up to the clearing point (*T_N–I_*), which is consistent with the presence of a skewed, SmC-like, cybotactic N phase (N_cyboC_) that extends over the entire N mesophasic range. Figure 5g–l (BCM **1c**) clearly shows the evolution from a high-temperature pattern, which is characterized by two diffuse meridional peaks, to the low-temperature four-spot pattern. This was interpreted as a structural transition (occurring at *T* − *T_N–I_* ≈ 6 °C, Figure 5i) between two different types of cybotactic N phases, namely the tilted N_cyboC_ at low temperature and, at higher temperature, the untilted N_cyboA_, i.e., a cybotactic N phase featuring an orthogonal layered, smectic A (SmA)-like, supramolecular structure [15].

The cybotactic model of the N phase of BCMs, originally proposed for mesogen **2** on the basis of XRD studies [11], has been confirmed over the years by several complementary experimental techniques including nuclear magnetic resonance (NMR) [19,20], dynamic light scattering (DLS) [21], dielectric spectroscopy [22,23,24,25], and second harmonic generation (SHG) [26], by molecular dynamics simulations [11,27,28] and, eventually, by direct imaging by means of cryo-transmission electron microscopy (Cryo-TEM) [29]. Unlike conventional (calamitic) LCs, where cybotactic order is primarily manifested as a pretransition effect restricted to the vicinity of an underlying Sm phase [18], the cybotactic order in BCMs persists over the entire N range up to the isotropic phase, and it does not exhibit critical behavior on approaching the N–SmC phase transition. In fact, it is regularly observed even in compounds not showing any Sm phase between the crystal and the nematic, as in the case of BCM **1a** [14]. In addition, when a Sm phase is present, comparison of the low-angle XRD patterns in the Sm and cybotactic N phases clearly shows an abrupt change of *d*-spacing and azimuthal intensity spreading across the Sm–N transition [16]. Overall, experimental evidence strongly suggests a picture of cybotactic order in BCMs—dynamic, very short-range fluctuations in the form of Sm-like density waves with the layers either titled (N_cyboC_) or orthogonal (N_cyboA_) relative to the director **n**—that is fundamentally different from the pretransition phenomena observed in conventional nematics. Nevertheless, other interpretations of BCM cybotaxis have been proposed, wherein the clusters are considered as more static entities permeating the N phase [30]. In this regard, preliminary X-ray photon correlation spectroscopy (XPCS) measurements performed by our group on BCM nematics did not reveal any dynamics within the time scale probed by the technique (10^−3^–10^0^ s), suggesting a sub-millisecond characteristic time for the cybotactic positional order.

### 2.3. Ferroelectric Switching

**Figure 6:**
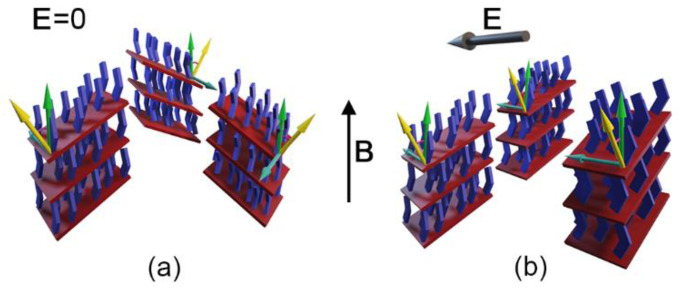
Alignment of cybotactic groups: (**a**) with no electric field **E** field applied, cluster dipoles are randomly oriented around the direction of the nematic director **n**, parallel to the magnetic field **B**; (**b**) upon application of an electric field orthogonal to **B**, cluster dipoles align along **E**. For each group, the green, light blue, and yellow arrows indicate the average direction of the long molecular axes, the average direction of molecular dipoles, and the layer normal, respectively. Adapted from [6], Copyright 2018, with permission from Elsevier.

Within cybotactic groups, BCM molecules may organize so that their individual dipoles, transverse to the molecular long axis, pile up coherently to produce a large overall polarization **P**, rather than canceling it out as occurs in a conventional N phase. This is because their bow shape coupled with a transverse dipole leads to a local packing favoring head-to-tail dipole arrangement and their consequent summing up. Each group exhibits local biaxial and possibly polar (ferroelectric-like) ordering due to the cooperative alignment of the molecules with their short axes and electric dipoles parallel to each other. Then, cybotactic groups can be regarded as the building blocks of a cluster N phase, similarly to how individual molecules are the constituent elements of a conventional molecular N phase. In the absence of an electric field **E**, the orientations of the cluster dipoles are randomly distributed around the molecular director **n**, and as a result, the macroscopic polarization *P* averages to zero: the N phase is macroscopically uniaxial and apolar (Figure 6a). Applying **E** above a certain threshold can align the cluster dipoles over macroscopic volumes, thus converting this non-polar phase into a biaxial and polar N phase (Figure 6b). Then, a net bulk polarization is induced, and its direction can be reversed by changing the sign of **E** [6,7,11]. In a conventional (molecular) N phase, this effect is prevented by thermal agitation, which largely overcomes the aligning action exerted by the field on individual molecules. By contrast, in a cybotactic (cluster) N phase, the field couples to an entire cluster of ordered molecules, so that its aligning action is magnified by a factor proportional to the average number of molecules per cluster and thus can prevail over thermal disorder. Once the polar state has settled, its switching can simply take place through the cooperative rotation of the molecules of each group about their long molecular axis and does not necessarily involve further rotation of the clusters, making the reorientation process relatively faster compared to the rotation of cybotactic groups as a whole.

**Figure 7:**
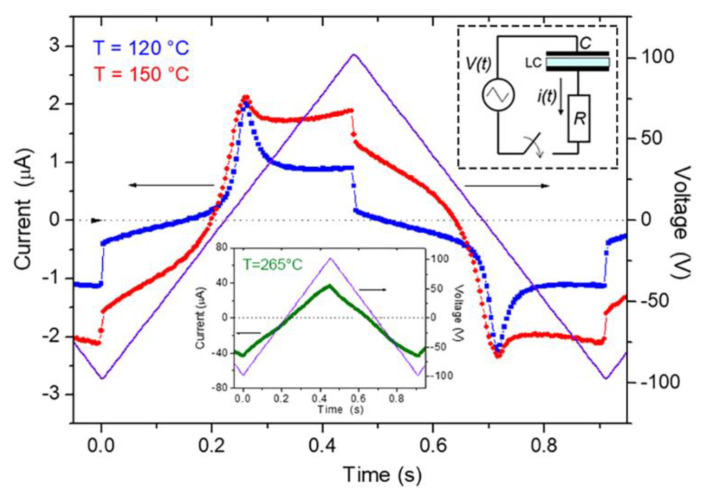
Repolarization current curves measured at three different temperatures, in the smectic C (SmC) (blue points), N (red points), and I (green points, in the inset) phases of BCM **2** (10 µm thick planar cell); the violet lines show the applied voltage. The upper inset shows the measurement circuit: *V*(*t*) is the applied triangular voltage waveform, *i*(*t*) is the circulating current, *C* is the capacitance of the LC cell, and *R* is the resistor across which the induced current is measured. Adapted from Francescangeli et al. [11]. Copyright© 2009 by WILEY-VCH Verlag GmbH & Co. KGaA. Reprinted by permission of WILEY-VCH Verlag GmbH & Co. KGaA.

The first experimental evidence of such a ferroelectric-like response to switching electric fields in a low molar mass nematic was reported in 2009 for BCM **2** of Scheme 1 [11] and later confirmed for other low molar mass bent-core nematics [6,31,32,33,34,35,36,37,38]. The study was carried out combining XRD with repolarization current measurements, electro-optical characterization and computer simulations. In particular, repolarization current measurements, performed by applying a low-frequency triangular voltage waveform across an LC cell and measuring the induced repolarization current (inset of Figure 7), revealed a characteristic ferroelectric response: in fact, the appearance of a single current peak per half-period, delayed with respect to the voltage reversal, is a well-known signature of a switching polarization (Figure 7) [6]. The fact that the repolarization peak was only present when the driving electric field exceeded a threshold value of about ± 7 V/µm and disappeared when the sample was heated to the isotropic phase was a further confirmation of the ferroelectric nature of the observed effect. In this way, polarization values as large as *P* ≈ 0.1 μC/cm^2^ could be measured, giving results that are in very good agreement with those of molecular dynamics simulations [11].

Beyond its fundamental interest, the ferroelectric behavior of BCM nematics could pave the way for a new generation of LC-based electro-optical devices. However, this technological potential is hampered by a few unfavorable features, such as the lack of reliable means to finely control the (potentially biaxial) BCM surface anchoring and the high onset temperature of the N phase of most BCMs. Addressing these issues through surface engineering and molecular design represent a major challenge of current research in the field of bent-core nematics [25].

### 2.4. Ferroelectric Switching in Liquid Crystal Polymers Based on Bent-Core Mesogens

**Figure 8:**
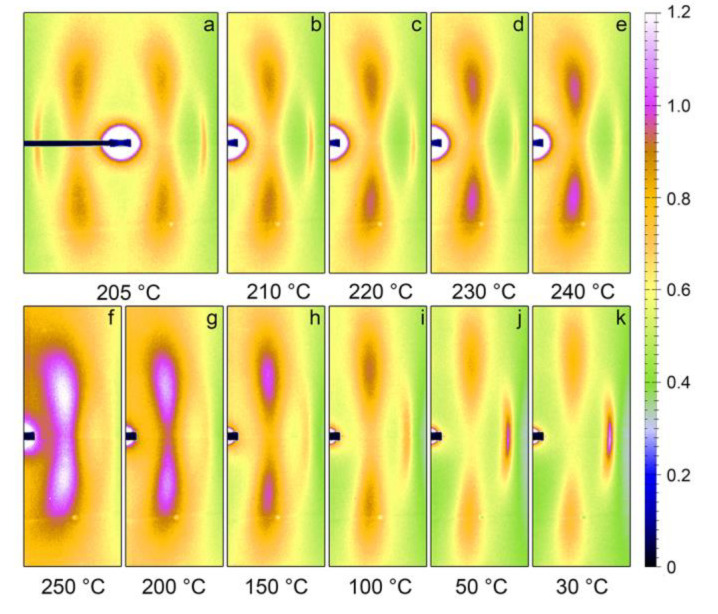
Low-angle XRD patterns of liquid crystal (LC) polymer **3** collected (**a**−**f**) on heating and (**g**−**k**) cooling the sample under a horizontal aligning magnetic field. Reprinted (adapted) with permission from [39]. Copyright 2014 American Chemical Society.

More recently, direct evidence of a ferroelectric-like switching response (upon application of an electric field of only 1.0 V/µm) was reported for the LC polymer **3** in Scheme 1, which is a main-chain polymeric counterpart of BCM **2** [39]. As summarized in Figure 8, the synchrotron XRD structural characterization unveiled the cybotactic structure of the N mesophase of this macromolecular compound, which allowed the ferroelectric switching response to be ascribed to a mechanism of cooperative rotation of the cybotactic clusters similar to that described above for the low molar mass parent BCM. Interestingly, repolarization current measurements on this compound provided polarization values up to *P* = 0.85 μC/cm^2^, which is almost one order of magnitude larger than the value measured for BCM **2** [11]. As cybotactic correlation lengths in polymer **3** are very similar to the ones measured in the low molecular weight parent compound **2**, the former’s larger polarization value is ascribed to the stronger orientational correlation imposed by intrachain bonds. Coupled with the chain-bond constraints, the cybotaxis in these macromolecular compounds results in maximized molecular correlations and hence in an enhanced ferroelectric response. This observation sets this class of materials among the most promising candidates in the search for the elusive biaxial and ferroelectric nematic phases. It was also found that the low tendency of these polymers to crystallize allows the cybotactic N phase to be supercooled down to room temperature (Figure 8k). While the switching behavior is lost in this low temperature state, supercooling could lead to a glassy N phase in which the biaxial and polar order are frozen at room temperature [39], with remarkable implications in the field of polymeric ferroelectric devices.

### 2.5. Field-Induced Phase Transitions

**Figure 9:**
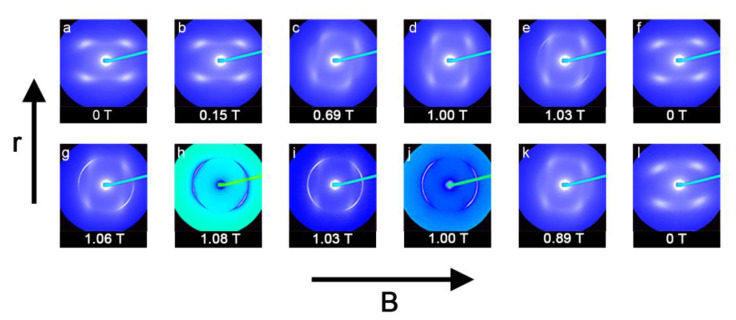
Low-angle XRD patterns measured for BCM **2** in a planar cell (anchoring axis **r** vertical) under a variable horizontal magnetic field **B** (field strength indicated below each pattern).

Figure 9 shows a sequence of low-angle XRD measurements performed on a 20 µm thick cell of BCM **2** in the N phase (*T* = 150 °C) under a horizontal magnetic field **B** (*y* axis in Figure 1), with the planar anchoring axis **r** set vertical (parallel to the *z* axis in Figure 1). In this configuration, the magnetic field competed with the mechanical field to orient **n** along mutually orthogonal directions. This is apparent in Figure 9a–d: starting from *B* = 0 (a), where the direction of **n** was imposed by surface anchoring (**r**), increasing *B* did not produce any effect until the Fréedericksz transition threshold field *B*_th_ was reached (0.15 T < *B*_th_ < 0.69 T), above which the four-spot pattern was rotated by 90 deg (c), as a result of the twist of **n** parallel to **B** in the bulk of the cell. A higher *B* intensity (*B* = 1 T) strengthened the orientation of **n** along **B** (d); however, further increasing *B* above 1 T triggered the transition of the sample to the SmC phase, as evidenced by the appearance of sharp arcs superimposed to the diffuse four-spot pattern (e). Subsequently, removing the magnetic field (f) returned the sample to the initial state of Figure 9a, i.e., in the N phase oriented by the mechanical field. Yet, rapid switching on the *B* field above threshold (g) and prolonged persistence at high field induced a full transition from the N to the SmC phase, as evidenced by the change of the four diffuse spots into four sharp and very intense arcs (h). Reducing the *B* field to 1 T did not immediately restore the N phase (i–j), which was recovered for *B* = 0.89 T with **n** parallel to **B** (k). Finally, switching off the magnetic field returned the sample to the initial undistorted N state, with **n** oriented by surface anchoring (l).

This sequence of patterns represents our first observation of an extraordinary effect, an N–SmC phase transition induced by a relatively low magnetic field as a consequence of the upward shift of the phase transition temperature (specifically the *T*_SmC-N_). To the best of our knowledge, this surprising behavior was never observed before in LCs to such an extent. In fact, magnetic and electric fields are normally able to macroscopically orient the **n** director, whereas their effect on the inherent thermodynamic properties of the phase (e.g., order parameter or transition temperatures) are very subtle, requiring substantial fields to be detected [2,40,41,42].

**Figure 10:**
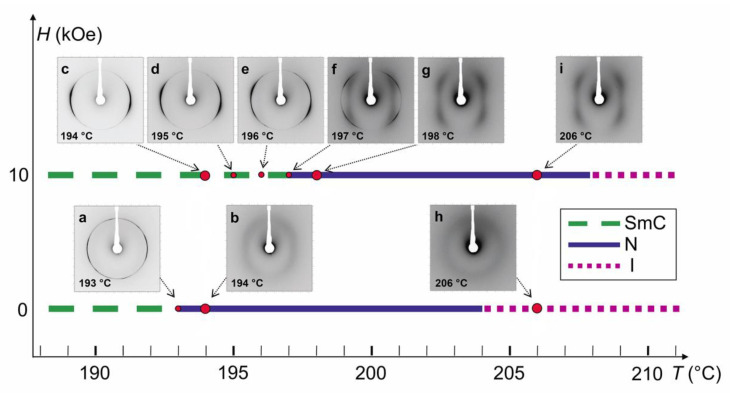
Phase diagrams of BCM **1b** at *H* = 0 kOe and *H* = 10 kOe. The insets show the low-angle XRD patterns at selected temperatures across the diagrams. Figure adapted with permission from [43]. Copyright 2011 by the American Physical Society.

To further investigate this unprecedented effect, we performed a series of XRD experiments aimed at studying the thermotropic phase diagram of BCM **1b** in the presence of magnetic (**H**) [43] or electric (**E**) [44] fields. Figure 10 compares the mesophase behavior of BCM **1b** at the zero *H* field with that measured at *H* = 10 kOe: the same mesophase sequence is observed in both cases, i.e., SmC–N_cybC_–isotropic (I), with a considerable shift (about 4 °C) of both the I–N_cybC_ and N_cybC_–SmC phase transition temperatures. This *giant H* field-induced temperature shift, never observed before as such, reflects the strongly enhanced susceptibility of the BCM N phase compared to conventional nematics and it is ascribed to the peculiar cluster structure of cybotactic nematics, in agreement with theoretical predictions [45]. In fact, in a molecular nematic, the single molecule interaction with the magnetic field has a negligible effect on the thermodynamic properties of the phase (orientational order parameter and transition temperatures). By contrast, in a cybotactic nematic, the coupling of the field with whole molecular clusters produces effects on the phase, which are enhanced by a factor proportional to the average number of molecules per cluster.

**Figure 11:**
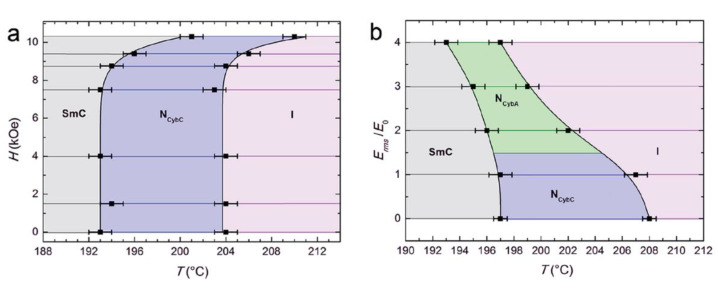
Temperature-field phase diagrams of BCM **1b** upon application of (**a**) magnetic and (**b**) electric fields (for the latter, 500 Hz root mean square field values are reported with *E*_0_ = 0.62 V/µm). Phase boundaries are drawn by interpolating the measured phase transition temperatures (square dots). Reproduced from [16] with permission from The Royal Society of Chemistry.

Similar effects on the mesomorphic phase behavior were observed upon the application of an electric field. The results of these studies are summarized in Figure 11a,b, showing the two-dimensional temperature-field phase diagrams of BCM **1b** in the case of magnetic and electric fields, respectively. It is apparent how relatively weak magnetic and electric fields, with no sizeable influence on a conventional N phase, can strongly modify the BCM phase diagram and induce isotherm phase transitions. Besides the relevance of this effect from a fundamental point of view, this cluster-mediated extraordinary sensitivity of BCM nematics to external fields is essential for the correct interpretation of their many unconventional properties and their potential technological exploitation.

### 2.6. Local Biaxial Order

Over the last few decades, the main driving force pushing the study of nematic BCMs was the expectation that these systems could represent the most promising candidates in the quest for the much sought-after biaxial N phase [46]. However, while some claims, essentially based on XRD [12,13] and NMR [47] measurements on BCMs **1b** and **1c**, seemed to confirm this prediction, it was later recognized that the seeming biaxiality of nematic BCMs was rather a result of the cybotactic nature of their N phase: while close packing constraints result in a biaxial (possibly polar) layered organization of the bent-core molecules on a nanometer length scale (the cybotactic groups), the whole phase is macroscopically uniaxial unless an external stimulus (electric, magnetic or mechanical) coherently aligns the cluster transverse axes along a common direction [7,8]. Thus, finding bent molecular structures able to promote biaxial ordering over a macroscopic length scale, yet preserving the characteristic fluidity of the N phase, is a major research goal in the field of liquid crystals [19].

**Figure 12:**
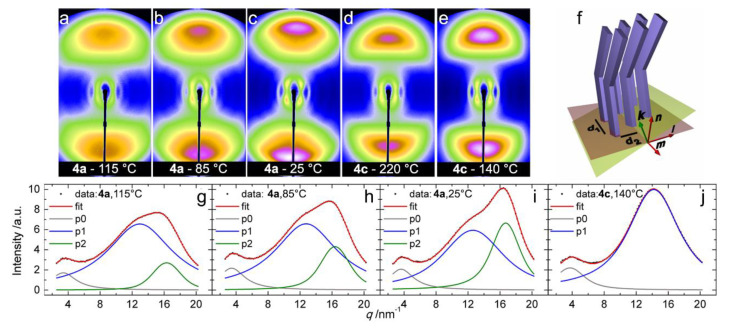
(**a–e**) Wide-angle XRD patterns for BCMs **4a** (**a**–**c**) and **4c** (**d**,**e**) at selected temperatures under a horizontal magnetic field **B** (‖ **n**); patterns (b,c,e) are in the supercooled N phase. (**f**) Schematic drawing of molecular packing within a tilted cybotactic cluster: **k** indicates the normal to the Sm plane (green), **n**, **m**, and **l** are the three orthogonal molecular directors; *d*_1_ and *d*_2_ are the intermolecular distances in the plane normal to **n** (purple). (**g**–**j**) Fit of the wide-angle equatorial (vertical) intensity profiles with two, p1 and p2, (**g**–**i**) or one, p1, (**j**) Voigt lineshapes (an additional small peak, p0, is added in the low-angle region to model peripheral scattering from the four-spot feature): (**g**) **4a** at 115 °C, (**h**) **4a** at 85 °C, (**i**) **4a** at 25 °C, and (**j**) **4c** at 140 °C. Reprinted (adapted) with permission from [49]. Copyright 2014 American Chemical Society.

Actually, initial XRD measurements performed on the cybotactic N phase of BCMs did not provide a direct proof of cluster biaxiality [8]. This feature was rather inferred from other experimental results (in particular, those provided by NMR studies [48]) and supported by the results of molecular dynamics simulations [11] as well as by comparison with the well-known biaxial and polar properties of BCM smectic phases [3]. The first XRD evidence of biaxial packing in the N phase of BCMs was reported for a class of laterally substituted compounds [49]. The addition of lateral substituents (methyl groups, in this case) to the main BCM structure is a well-known strategy to lower the nematic temperature range toward room temperature. It was found that the trimethylated BCMs **4a–b** in Scheme 1 showed a significant reduction of the nematic onset temperature, from 200 °C in the unsubstituted parent compound (**1a** in Scheme 1) to more treatable 124 °C and 144 °C temperatures in BCMs **4a** and **4b**, respectively [50]. In addition, the N phase of the latter compounds could be supercooled down to room temperature into a highly viscous metastable N phase. Upon XRD investigation, the N phase of methylated compounds showed the characteristic low-angle four-spot pattern of cybotactic BCMs. However, trimethylated BCMs **4a** and **4b** also showed a peculiar wide-angle diffraction pattern (Figure 12a–c), clearly consisting of two broad transverse reflections, which were largely superimposed but still resolvable, corresponding to transverse *d*-spacings differing from each other by about 1 Å, i.e., *d*_1_ ≈ 3.8 Å, and *d*_2_ ≈ 4.9 Å (Figure 12g–i) [49]. This highly unconventional diffraction pattern, never reported before in BCMs, is strictly related to the specific substitution pattern of compounds **4a** and **4b**. In fact, the same splitting of the wide-angle reflection was still present when the BCMs entered the isotropic phase [51], and it was later reported for the N phase of other trisubstituted compounds, either based on a slightly different core (an oxazole instead of an oxadiazole) [52] or featuring a halogen substituent instead of a methyl on the inner phenyl ring [53]. By contrast, as shown in Figure 12, both the unsubstituted parent compound **1a** and monomethylated derivates such as BCMs **4c** and **4d** exhibited the usual wide-angle diffraction pattern (Figure 12d,e), consisting of a single broad reflection pointing at a transverse *d*-spacing of approximately 4.5 Å, intermediate between *d*_1_ and *d*_2_ (Figure 12j) [49].

The observation of two different *d*-spacings in the plane transverse to **n** can be rationalized by assuming a biaxial packing of the LC molecules, with *d*_1_ representing the average spacing between *π*-stacked aromatic rings and *d*_2_ being associated to their in-plane distance (Figure 12f). Unfortunately, XRD measurements are unable to provide an estimate of the spatial extent of such biaxial ordering, whereas optical measurements performed of BCMs **4a** and **4b** have demonstrated the uniaxial nature of their N phase [54]. It must be concluded that XRD data only provide evidence of local biaxial ordering, possibly extending over the typical length scale of cybotactic groups. Nevertheless, the fact that such an XRD feature is only observed in a very specific class of laterally substituted BCMs reveals the peculiarity of trimethylated compounds, which clearly manifest an enhanced tendency toward biaxial order. Within this framework, understanding the relationship between this unconventional behavior and the details of the molecular structure could provide precious hints for the engineering of BCMs exhibiting spontaneous and macroscopic biaxiality. To pursue this goal, we have recently extended our investigation to different substitution patterns (i.e., di- and tetra-methylated derivatives). It was found that the splitting of the wide-angle XRD pattern is not necessarily associated to the ability of the N phase to be supercooled to room temperature: while the presence of methyl groups on the outer phenyl rings is crucial to the former, the latter is directly related to the symmetry of the substitution pattern (with the lack of symmetry hampering the crystallization) [55]. Apparently, the presence of methyl groups on the outer phenyl rings, probably interacting with the nearby carbonyl oxygens, strongly influences the molecular conformation and mobility, with a direct effect on the molecular packing in the transverse direction.

## 3. All-Aromatic Liquid Crystals

### 3.1. Materials and Methods

**Scheme 2:**
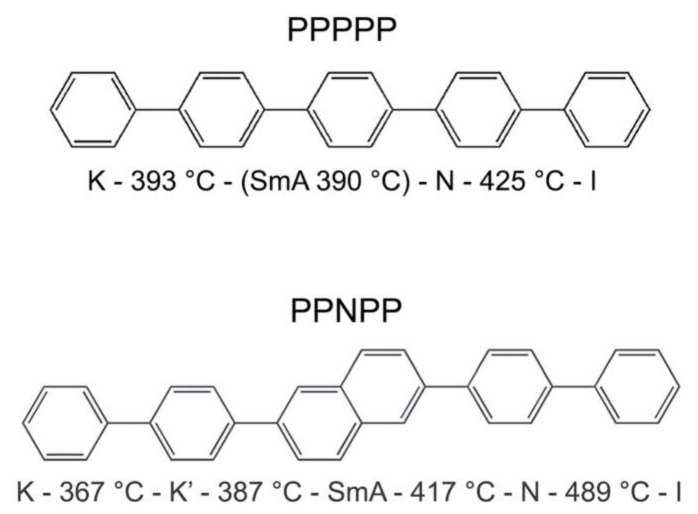
Chemical structure and phase diagram of investigate all-aromatic mesogens.

Rod-like all-aromatic compounds represent prototypical nematogens, an ideal benchmark for scientists interested in relating theories of N order to experiments. In particular, all-aromatic mesogens such as *p*-quinquephenyl (PPPPP, Scheme 2) and 2,6-biphenyl naphthalene (PPNPP, Scheme 2) exhibit a high-temperature enantiotropic N phase, extending well above 400 °C. Their rigid and cylindrically symmetric structure, devoid of polar groups and flexible chains, represent the closest embodiment of the highly idealized rod-like mesogen at the basis of theories of the N phase. The investigation of these prototypical nematogens is expected to contribute to unveil the basic mechanisms underlying the onset of the short-range positional order in the N phase.

**Figure 13:**
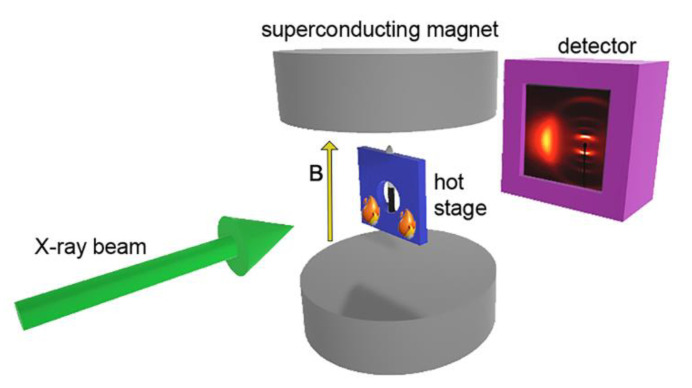
Experimental setup for XRD measurements on all-aromatic mesogens under a maximum magnetic field of 4 T. The hot stage allowed measurements up to 600 °C with a stability better than ± 0.5 °C.

In spite of the great fundamental interest, the literature provides very few experimental studies on this class of nematics [56,57,58], which is mainly due to the challenge of performing measurements at temperatures exceeding 400 °C on highly volatile samples. In order to gain insights into the relationships between molecular shape, mesophase properties, and theoretical predictions, synchrotron radiation was recently used for the first time to probe the nanoscale structure of the high-temperature N phase of these compounds [59]. In these experiments, quartz capillary samples sealed to prevent sublimation were mounted on a temperature-controlled hot stage allowing the insertion of a static **B** field (up to 4 T, generated by a superconducting magnet [10]) orthogonal to the X-ray incident beam (Figure 13). The hot stage was specifically designed to work up to 600 °C with a thermal stability better than ± 0.5 °C.

### 3.2. Positional Order

**Figure 14:**
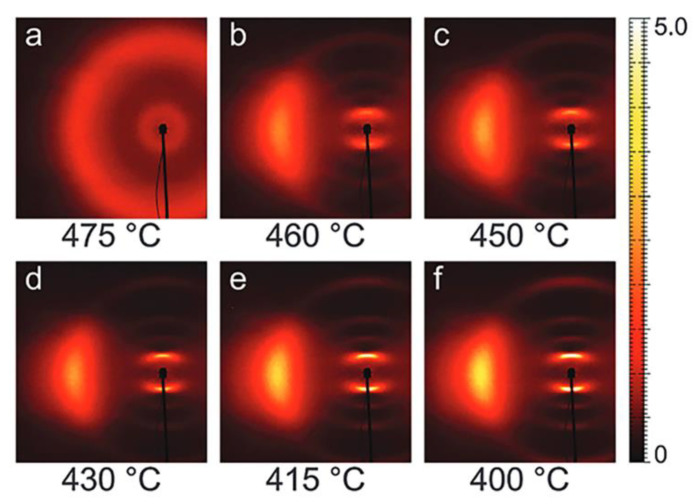
2,6-biphenyl naphthalene (PPNPP) wide-angle diffraction patterns measured on cooling from the isotropic melt upon application of an aligning magnetic field (2.2 T, vertical). Only the left wide-angle feature is shown because of the pattern symmetry. Reproduced from [59] with permission from The Royal Society of Chemistry.

The experimental results revealed unconventional features in the XRD pattern of the oriented N phases of PPNPP, which were consistent with an unexpected high level of molecular clustering (cybotaxis) that persists across their entire wide N range. Figure 14 shows a representative sequence of PPNPP diffraction patterns in the aligned N phase, which were taken upon cooling from the isotropic phase. The sample entered the N phase at 470 °C, as indicated by the abrupt change of the diffuse ring characteristic of the isotropic phase (Figure 14a) into the anisotropic diffraction pattern typical of an aligned nematic (Figure 14b–f). The latter is characterized by two relatively intense low-angle reflections centered on the meridional direction (‖ **B**), by a sequence of weaker diffraction features spanning the intermediate to wide-angle region along the meridional direction, and by a pair of diffuse reflections centered on the equatorial direction (⊥ **B**). On lowering the temperature below 400 °C, the sample entered the SmA phase, identified by the nonlinear increase of intensity and concomitant width narrowing of the low-angle meridional reflections.

**Figure 15:**
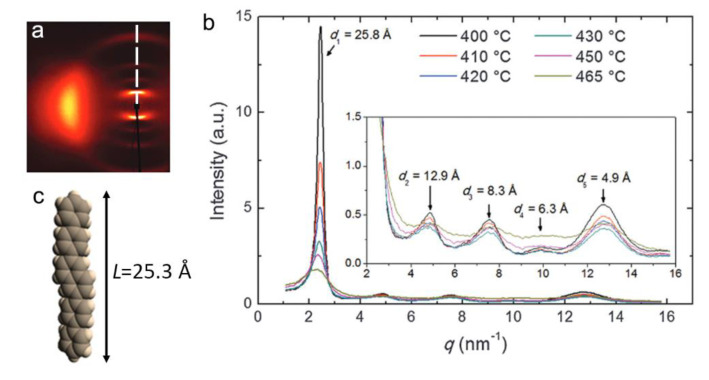
(**a**) Representative wide-angle diffraction pattern of PPNPP showing the measurement direction (white dashed line) for the meridional (‖**n**) intensity profiles shown in the subsequent panel. (**b**) Meridional intensity profiles of PPNPP at selected temperatures (the inset shows a close-up of the higher *q* reflections). Arrows associate each peak to the corresponding *d*-spacing. (**c**) PPNPP molecular model and estimated molecular length. Adapted from [59] with permission from The Royal Society of Chemistry.

Structural parameters relevant to the molecular arrangement in the N phase were found from a detailed analysis of the above XRD patterns. The intensity profile along the meridional direction (Figure 15a, white dashed line) is shown in Figure 15b. The scattering vector of the main peak, due to the low-angle reflection, is *q*_1_ = 2.44 nm^−1^, which is equivalent to a *d*-spacing *d*_1_ = 2*π*/*q*_1_ = 25.8 Å. This value, indicating the average distance between adjacent molecules in the longitudinal direction, agrees very well with the mesogen length *L* = 25.3 Å estimated by molecular models (Figure 15c).

**Figure 16:**
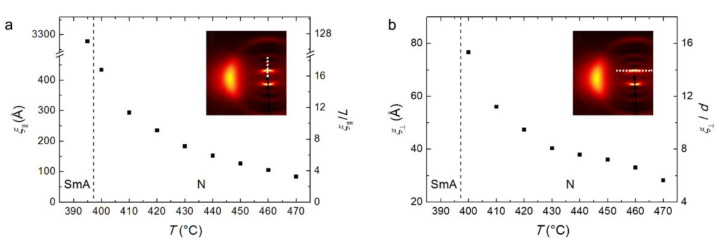
Cybotactic order correlation lengths in PPNPP as a function of temperature: (**a**) longitudinal correlation length, *ξ*_‖_, and its ratio with the molecular length *L*; (**b**) transverse correlation length, *ξ*_⊥_, and its ratio with the transverse intermolecular distance *d*. Correlation lengths were obtained from the full width at half maximum (FWHM) of the low-angle peak measured along the cuts shown in the two insets. Panel (**a**) adapted from [59] with permission from The Royal Society of Chemistry.

The relatively sharp nature of the meridional low-angle diffraction peak is indicative of an unconventional degree of the longitudinal positional order for the N phase. Such evidence, together with the absence of low-angle peak splitting (i.e., absence of the four-spot pattern) points at a cybotactic N phase of the normal (orthogonal) type, featuring SmA-like stratification within clusters of mesogens. Following the procedure described in Ref. [59], the longitudinal position correlation length *ξ*_‖_ was estimated from the meridional FWHM of the peak (inset of Figure 16a) using Hosemann’s paracrystalline model [60]. The results, shown in Figure 16, reveal a surprisingly high level of positional longitudinal order, with the corresponding correlation length increasing from a conventional value of approximately 3*L* just below the isotropization temperature to the significantly larger value of approximately 17*L* on approaching the SmA–N transition. On further cooling the sample, the peak width becomes comparable to the experimental resolution, which is a signature of the long-range correlation length characterizing the SmA phase. Such large longitudinal correlation lengths, exceeding the typical value of approximately 5*L* [60] over a large part of the N temperature range, are unusual in LCs and suggest a strong propensity of PPNPP to stratify within the N phase, i.e., to exhibit normal, SmA-like, cybotactic order. An estimate of the transverse extension of a layered order could be obtained from the FWHM of the low-angle peak measured along the transverse (equatorial) direction (inset of Figure 16b). Adopting the same approach used to evaluate *ξ*_‖_, we obtained the values of the transverse correlation length *ξ*_⊥_ shown in Figure 16b. They range between approximately 6*d* (at 470 °C) and approximately 15*d* (at 400 °C), *d* ≈ 5 Å being the transverse intermolecular distance estimated from the scattering vector associated to the maximum of the broad equatorial reflection (*q* approximately 12.5 nm^−1^).

Based on these results, the average cluster volume evaluated via the longitudinal, *D*_‖_ ≈ 3*ξ*_‖_, and transverse, *D*_⊥_ ≈ 3*ξ*_⊥_, cybotactic clusters size turns out to be remarkably higher than that found in the cybotactic N phase of BCMs, revealing a surprisingly propensity for molecular layering in the N phase. This finding represents an unexpected result, as PPNPP does not possess the features conducive to molecular layering inherent to cybotactic ordering in BCMs, neither in its shape nor chemical constitution: the PPNPP architecture is devoid of diverse chemical components (aromatic versus aliphatic molecular segments) and polar groups, which are believed to be essential for smectic layering in low molar mass thermotropic mesogens. This result has important fundamental implications, as it appears to suggest the universal nature of cybotactic order, which is thus not specific to BCMs.

### 3.3. Orientational Order

**Figure 17:**
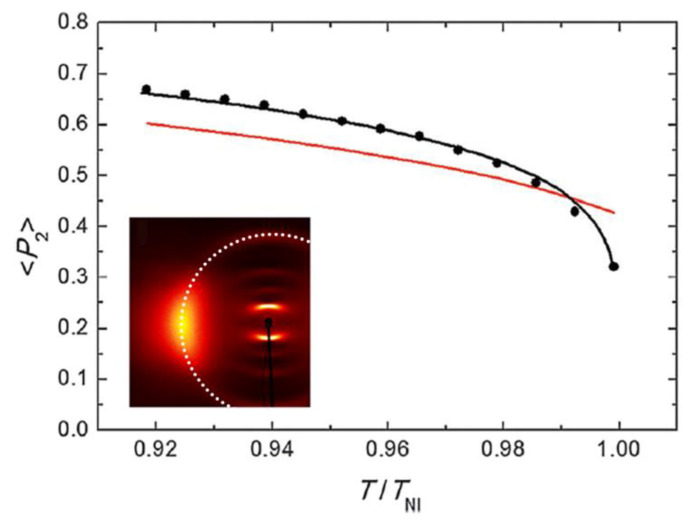
Orientational order parameter <*P*_2_> dependence on the reduced temperature *T*/*T*_NI_: experimental values (black dots) fitted by the Haller equation (black line) and compared to the Maier–Saupe (MS) theoretical prediction (red line). <*P*_2_> values were obtained by analyzing the azimuthal intensity profile of the wide-angle reflection measured along the dotted line shown in the inset. Adapted from [59] with permission from The Royal Society of Chemistry.

The orientational order parameter <*P*_2_> of the cybotactic N phase of PPNPP was experimentally determined to investigate to which extent it compares with that typically found in conventional calamitic mesogens. This was obtained from the azimuthal broadening of the diffuse wide-angle reflection (Figure 14b–f), following the classical method described in [61]. The model assumes that the equatorial wide-angle reflection is due to transverse diffraction from groups of few quasi-parallel molecules. Thus, the experimental broadening of this diffraction feature can be mathematically related to the orientational distribution function of such groups, which in turn can be considered a good approximation of the single molecule orientational distribution function. By assuming a Maier–Saupe (MS) form for the orientational distribution function, fitting the azimuthal intensity profiles of the wide-angle feature (measured along the dotted line shown in the inset of Figure 17) provided the values of <*P*_2_> shown in Figure 17 as a function of the reduced temperature *T*/*T*_NI_ (*T*_NI_ being the clearing point). In the figure, the experimental values are well fitted by a Haller-type equation $\langle P_{2}\rangle ={\left(1-\frac{T}{T_{\mathrm{NI}}}\right)}^{\beta }$, with *β* = 0.165 ± 0.002, and contrasted with the predictions of the MS theory for nematic order. The comparison is relevant, as theory predicts significant deviations from the universal MS curve for the orientational order parameter of a cybotactic N phase [62]. In our case, we observed <*P*_2_> values larger than the MS curve over most of the N temperature range: while this discrepancy is comparable to the estimated accuracy of the measurement technique [61], it could also be a significant effect of the high level of cybotactic order discussed above. On the other hand, a steep decrease of <*P*_2_> is observed on approaching the clearing point, contrasting with the universal value <*P*_2_> = 0.43 predicted by the MS theory at *T*_NI_. This latter behavior is often observed in experiments measuring the temperature dependence of <*P*_2_> in nematic LCs. In particular, it has been reported for the orientational order parameter of another all-aromatic compound, PPPPP, which was evaluated by diamagnetic susceptibility [56], NMR [57], and XRD measurements [58]. While it could just reflect macroscopic orientational fluctuations of the molecular director occurring at high temperature in the vicinity of *T*_NI_, in our case this effect is expected to be largely prevented by the use of a relatively strong aligning magnetic field of 2.2. T. Overall, these measurements suggest significant deviations from the conventional MS behavior, whose unequivocal confirmation would require additional investigations with the utmost accuracy.

Interestingly, a similar temperature dependence of the orientational order parameter was predicted by molecular dynamics simulation for the related all-aromatic mesogen PPPPP [63]. These findings prompted us to investigate the N phase of PPPPP to determine if cybotactic order is a unique feature of PPNPP or if it is present in other all-aromatic mesogens, thus calling into question the long-held picture of the N phase as made of independent diffusing mesogens merely featuring an orientational order. In a recent study, we have compared a high-resolution synchrotron XRD structural characterization of both PPNPP and PPPPP [64]. New insights into the positional short-range order in the N phase of all-aromatic mesogens from the nano- to the meso-scale are observed. Preliminary results show that cybotactic order persists in the N phase of PPPPP as well as PPNPP, thereby suggesting that cybotaxis is a general property of the N phase of all-aromatic mesogens and possibly of all thermotropic LCs. This observation has wide ranging, fundamental implications for the current picture of the N phase as a molecular liquid without short-range positional order.

## 4. Polymer Dispersed Liquid Crystals

### 4.1. Introduction

PDLCs are composite materials consisting of droplets of low molar mass LC randomly dispersed in a polymeric matrix [65,66,67,68]. The droplet sizes may vary from the sub-micrometer scale up to tens of microns, with droplets in the range of 0.5–5 µm being the most common. The droplets are randomly distributed in the polymer, and when their size is close to the visible wavelength, they produce a strong scattering of the incident light. A large variety of structures are possible, depending on the concentration, nature, and properties of the polymer and the LC. These optoelectronic materials exhibit unique linear and nonlinear optical properties [66,67] that make them suitable for a number of technological applications, such as smart windows, novel flat-panel displays, optical switches and modulators, sensors, and holographic films [69,70].

The principle of operation is quite simple and is based upon the field-controlled light scattering from the LC microdroplets. In fact, while the polymeric matrix acts as an isotropic solid binder, the droplets exhibit a strong optical anisotropy that depends on the orientation of the LC therein. Such orientation can be easily controlled by an external electric or magnetic field; in turn, this affects the refractive index mismatch between the LC droplets and the polymeric matrix and hence the PDLC film optical transmittance. In particular, applying an electric (driving) field of the order of 10^4^ V/cm across the PDLC film can switch the film from an opaque highly scattering state to a transparent highly transmissive state. Sub-millisecond switching times are possible.

**Figure 18:**
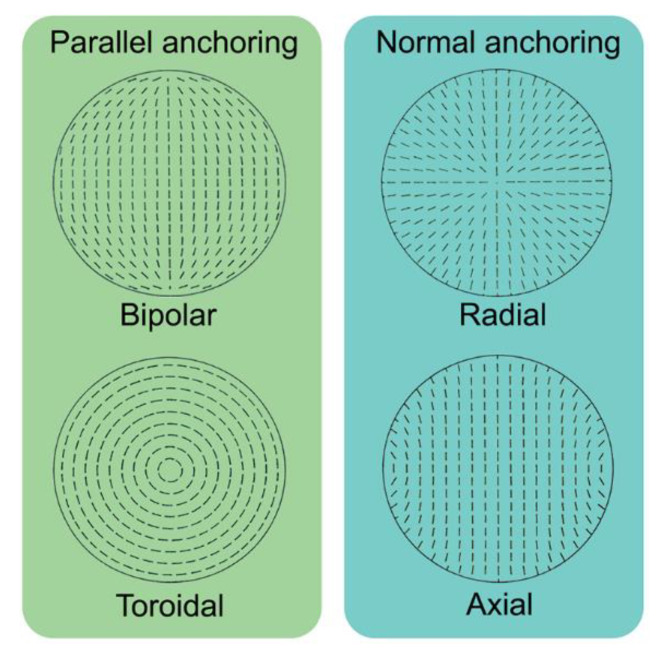
Schematic representation of four possible director configurations in a droplet: bipolar, toroidal, radial and axial.

Beyond the scope of applications, in the last decades, the interest in PDLCs has considerably stimulated fundamental research concerning the polymerization-induced phase separation process used for their preparation [71], their dielectric and optical properties [72,73], the reorientational dynamics of the LC inside the droplets [74], the dependence of the measured macroscopic parameters (e.g., switching time, driving voltage, etc.) on the shape and distribution of the droplets, and especially the unusual physical properties exhibited by the LC when confined to small cavities [75]. In fact, the large surface-to-volume ratio characterizing the PDLC morphology results in a variety of unconventional effects such as changes in the nature of the phase transitions and specific director configurations. Determining the details of the nematic director configuration within the droplets is of primary importance in the study of PDLCs. When the droplet sizes are of the order of a few microns, the critical interplay between the aligning action induced by anchoring at the boundary surface and the strong elastic deformation inside the volume results in a variety of director configurations (Figure 18), including point and line defects as well as twisted structures [65]. Temperature changes or external fields can induce transitions from one configuration to another, and these transitions can in turn be used to obtain a measure of the surface anchoring strength [76].

### 4.2. Materials and Methods

The most conventional experimental tools used to determine the nematic director configuration within LC droplets are polarized optical microscopy (POM) and NMR spectroscopy. POM is easier to perform but is limited to droplet sizes greater than a few microns. ^2^H-NMR is in general more powerful [77]; however, it requires specifically deuterated nematic probe molecules and provides only averaged information over a large number of droplets. In addition, to prevent significant distortion by the magnetic field, the droplet radius is requested to be *R* < 0.5 µm.

**Figure 19:**
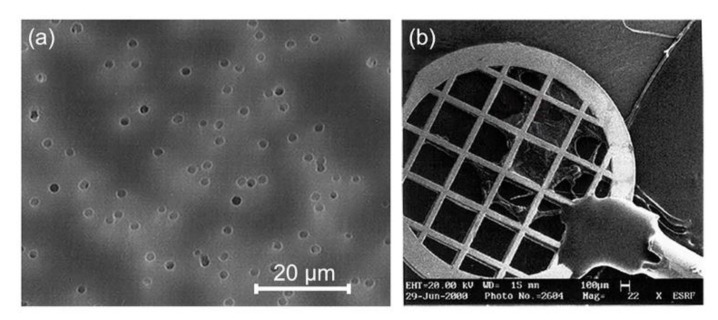
(**a**) SEM micrograph of a section of the investigated polymer-dispersed liquid crystal (PDLC) sample; (**b**) PDLC film on an electron microscopy copper grid.

In a pioneering experiment performed in the early 2000s, O. Francescangeli et al. [78] demonstrated for the first time the effectiveness of synchrotron X-ray microdiffraction (*μ*-XRD) as a new experimental tool to probe LC ordering and director-field configuration within single droplets of PDLCs. A micro-focused X-ray beam was used in transmission geometry and diffraction patterns from single droplets of an ultrathin PDLC film were collected and analyzed. The samples were prepared via thermally initiated polymerization-induced phase separation starting from a mixture of the epoxy resins EPON815 (by Shell, 25.4% in weight) and MK107 (by Wilmington, 7.1%) with the hardener Capcure 3-800 (by Miller Stephenson, 32.5%) and the nematic LC E7 (by Merck, 35%). The mesophase sequence of E7 is crystal (K)—nematic (N)—isotropic (I) with transition temperatures *T*_KN_ = 263 K and *T*_NI_ = 334 K. This mixture gives rise to LC droplets with bipolar configuration [65,66], the preferred configuration in the case of strong parallel molecular anchoring. This configuration is characterized by cylindrical symmetry where the symmetry axis is defined by two surface-point defects lying at the opposite ends of the droplet surface [79]. Ultrathin films of the PDLC samples were sliced out by means of a liquid nitrogen-refrigerated microtome. The thickness of the film was fixed to 5 µm in order to reduce the probability for the probing X-ray beam to cross more than one droplet. The analysis of a section of the sample by means of scanning electron microscopy revealed a narrow distribution of the spherical droplet size, with an average droplet diameter of 2.0 ± 0.1 µm (Figure 19a).

**Figure 20:**
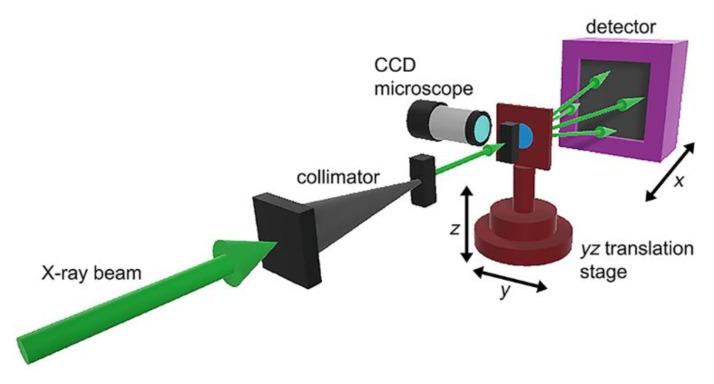
Schematic drawing of the scanning synchrotron X-ray microdiffraction (*μ*-XRD) setup.

The diffraction experiments were carried out using the scanning small-angle/wide-angle microdiffraction setup of the ID13 microfocus beamline at the ESRF (Figure 20). A monochromatic beam (*λ* = 0.784 Å or *λ* = 1.109 Å) of 2 µm diameter (2 μrad divergence) produced by a tapered-glass capillary X-ray optics was used. The PDLC film sample was supported by an electron microscopy copper grid (Figure 19b), which was mounted on a high-resolution (<1 µm) translation stage. A charge-coupled device (CCD) microscope was used to select areas of interest on the sample (Figure 20). The data were collected at room temperature. The resolution in the scattering angle was Δ(2*θ*) = 3 × 10^−2^ degrees. The selected region was mapped in steps of 2 µm in both the horizontal and vertical directions. Different areas of the sample were investigated.

### 4.3. Single Droplet Director Configuration

As the polymer matrix is amorphous and isotropic (as confirmed by *μ*-XRD measurements on thin films of samples after removing the LC by evaporation under vacuum), whereas bipolar droplets possess LC ordering and anisotropic director configuration, it was possible to discriminate between the scattering from a droplet and that from the polymer binder in all cases except when the droplet director **N** (i.e., the average direction of the nematic director **n** within a droplet) was parallel to the incident beam. In fact, when the X-ray beam intercepted an LC droplet, an anisotropic pattern featuring a pair of weak and diffuse low-angle reflections close to the beam stop was observed. These reflections are a characteristic diffraction feature of aligned nematics and are caused by the short-range positional order in the direction parallel to **n**. By contrast, isotropic patterns without any low-angle signal were observed for the polymer matrix.

**Figure 21:**
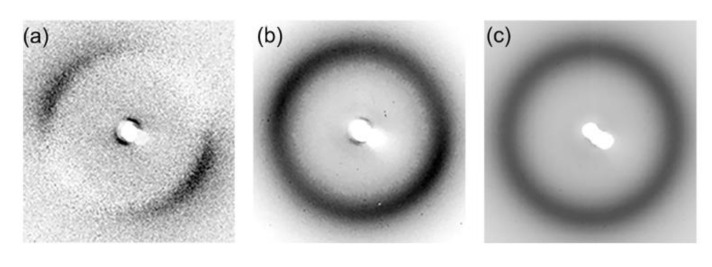
(**a**) *μ*-XRD pattern from a single spherical droplet after subtraction of the isotropic background due to scattering from the polymer; (**b**) corresponding *μ*-XRD pattern before background subtraction; (**c**) background diffraction pattern from the polymer binder. The pattern in (**a**) is obtained subtracting the pattern (**c**) from (**b**). Adapted from [78] with the editor’s permission.

The anisotropic diffraction pattern from LC droplets was more evident after background subtraction. Figure 21a shows a representative example of the *μ*-XRD pattern of a single LC droplet as obtained after subtracting from the measured pattern (Figure 21b) the background isotropic diffraction pattern due to the polymeric matrix surrounding the selected droplet (Figure 21c). The anisotropy of the pattern in Figure 21a indicates a molecular director field configuration possessing cylindrical symmetry and is similar to the typical diffraction patterns observed for axially oriented nematics [79]. In the low-angle region, the dominant feature is the pair of diffuse meridional (**q** ‖ **N**) reflections centered at *q* = 0.218 Å^−1^, corresponding to a spacing *d* = 2*π*/*q* = 28.8 Å and arising from longitudinal correlations in the molecular arrangement. The two diffuse wide-angle crescents centered on the equatorial line (**q** ⊥ **N**) at *q* ≈ 1.4 Å^−1^ are associated with the short-range (liquid-like) transverse positional order of the molecules and correspond to an average molecular distance *d* ≈ 4.5 Å.

**Figure 22:**
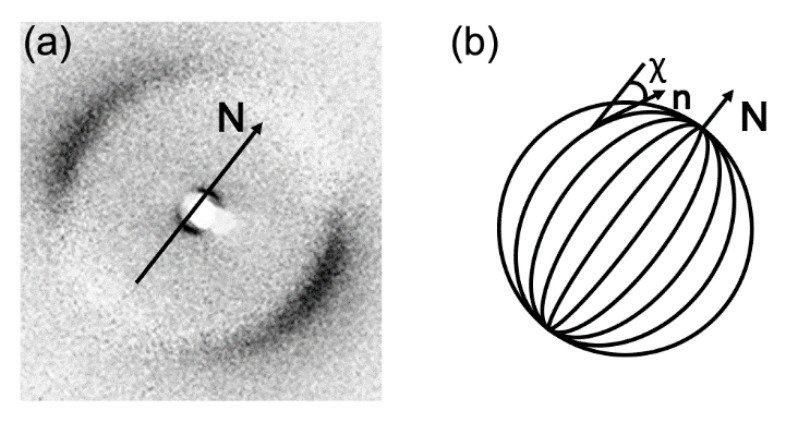
(**a**) Background subtracted *μ*-XRD pattern from a single bipolar droplet with droplet director **N** and (**b**) corresponding schematic representation of the bipolar configuration. Adapted from [78] with the editor’s permission.

**Figure 23:**
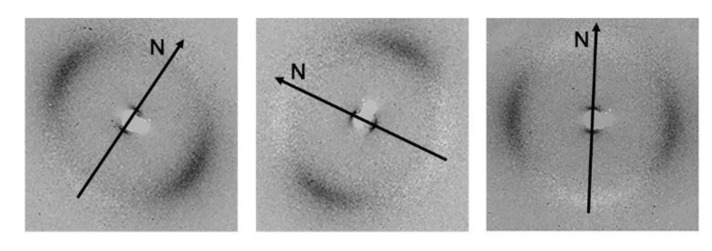
Background subtracted *μ*-XRD patterns of single droplets exhibiting different orientations of the droplet director **N**. Adapted from [78] with the editor’s permission.

**Figure 24:**
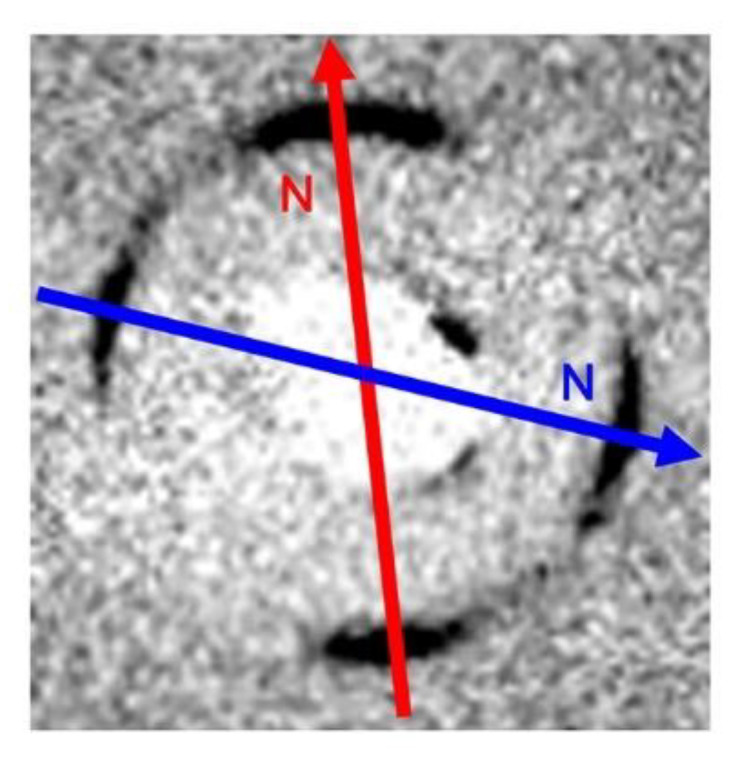
Low-angle region of *μ*-XRD pattern recorded in a sample point where the X-ray beam crosses two distinct droplets with different axial orientations pointed out by the blue and red versors.

Thus, the droplet director, **N**, is aligned with the low-angle reflections, i.e., the meridional line (Figure 22a,b). The symmetry of this pattern around the equator ensures that the droplet director lies in the plane orthogonal to the incident beam or very close to it. Figure 23 shows the *μ*-XRD patterns of distinct single droplets in the sample that exhibit different orientations of the droplet director **N**. As a further example of the potential of the technique, Figure 24 shows the low-angle region of a *μ*-XRD pattern exceptionally recorded in a point of the sample where the X-ray beam crossed sequentially two distinct droplets with different axial orientations.

### 4.4. Droplet Order Parameter

The apparent azimuthal broadening of both the meridional and equatorial reflections points to a distribution of angular orientations of the coherent scattering nematic domains relative to the droplet director **N**. This distribution reflects the curvature of the nematic director field lines in the bipolar droplet and is inherently connected to the specific configuration assumed by the director field inside the droplets (Figure 22b). The azimuthal intensity profile of the diffuse wide-angle crescents was used for the first time [78,80] to determine experimentally the orientational distribution function of the nematic director inside the droplet and to calculate the droplet order parameter.

The concept of droplet order parameter was originally proposed within a model formulated to describe the electrooptical response of PDLCs [81]. This approach introduces a hierarchy of order parameters that is suitable to describe the orientational order on different length scales. The nematic within a droplet is assumed to possess a local director configuration and order parameter; then, a droplet director **N** and a droplet order parameter *S*_D_ are defined representing the average orientation of the nematic director **n**

(1) $$S_{D}={\langle \frac{3{\left(N\cdot n\left(r\right)\right)}^{2}-1}{2}\rangle }_{V_{D}}={\langle P_{2}\left(N\cdot n\left(r\right)\right)}_{V_{D}},$$

where *P*_2_(**N** · **n**(**r**)) is the second-order Legendre polynomial, and the average is calculated over the droplet volume *V*_D_.

**Figure 25:**
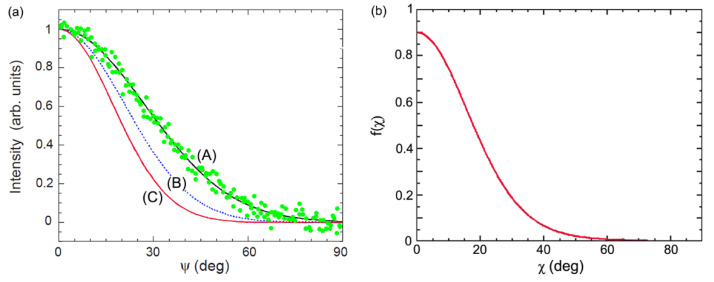
In panel (**a**) are shown: (A, green dots and black line) azimuthal intensity profile (at constant *q* = 1.4 Å^−1^) of the diffuse wide-angle crescent in a single droplet *μ*-XRD pattern with the green dots and the full black line representing the experimental data and their best fit by a Gaussian lineshape, respectively; (B, dotted blue line) theoretical azimuthal intensity profile that should be obtained with **n** parallel to **N** everywhere within the droplet; (C, red line) deconvolution of curves (A) and (B). All the curves are normalized to their maximum value. The panel (**b**) shows the orientational distribution function of the nematic director inside the bipolar droplet. Adapted from [78] with the editor’s permission.

The curve A in Figure 25a shows the azimuthal intensity profile (at constant *q* = 1.4 Å^−1^) of the diffuse wide-angle equatorial reflections. The azimuthal spread of these diffraction features reflects two distinct causes: (1) the curvature of the **n** director field lines inside the droplet and (2) the thermal orientational disorder of the molecules that results in a distribution of molecular orientations around the local average direction **n**(**r**). The latter effect is always present, even for an ideal uniform configuration with **n** parallel to **N** at each point inside the droplet (i.e., *S*_D_ = 1). In that case, the azimuthal intensity profile at constant *q*, *I*_0_(*ψ*) (*ψ* being the azimuthal angle on the detector) is related to the orientational distribution function of the molecules, *f*(*β*), as follows

(2) $$I_{0}\left(\psi \right)=\int_{\beta =\psi }^{\pi /2}f\left(\beta \right)\sec^{2}\psi {\left(\tan^{2}\beta -\tan^{2}\psi \right)}^{-1/2}\sin\beta d\beta ,$$

where the azimuth *ψ* is measured relative to the equatorial axis and the distribution function *f*(*β*) is defined such that 2*πf*(*β*)sin*β*d*β* gives the fraction of molecules that have their long axes at an angle between *β* and *β* + d*β* with respect to the director **n**.

The function *I*_0_(*ψ*) represents the intrinsic spreading associated to the thermal molecular disorder and, following the lines described in [78,80], it can be determined via Equation (1) assuming a Maier–Saupe mean-field distribution for *f*(*β*), i.e., *f*(*β*) = exp[*m*cos^2^*β*]/4*πZ*, where *Z* is a normalization constant, *m* = 3(*US*/*k*_B_*T*)/2, *U* being the orientational potential energy, and *S* = <3 cos^2^*β* – 1>/2 being the nematic order parameter. The room-temperature value of *m* = 4.45 was determined by imposing that the nematic order parameter equals the value 0.6 experimentally measured in bulk nematic E7. Then, the numerical integration of Equation (2) allowed calculating *I*_0_(*ψ*), and the result is shown in Figure 25a (dotted line B).

In the case of a general configuration with curved director field lines, the contribution of elementary domains having different average orientation **n** must be considered. If one assumes that the LC order parameter *S* is not appreciably affected by the confinement and is the same at each point in the droplet, the azimuthal spread of the intensity profile can be expressed through the convolution integral [78]:

(3) $$I\left(\psi \right)=\int_{0}^{\pi }I_{0}\left(\psi -\alpha \right)G\left(\alpha \right)d\alpha ,$$

where *I*_0_(*ψ*) is given by Equation (2) and the function *G*(*α*), accounting for the azimuthal intensity spreading due to the orientational distribution of **n**, can be expressed by means of an equation that is formally equivalent to Equation (2):

(4) $$G\left(\alpha \right)=C\int_{\chi =\alpha }^{\pi /2}f_{D}\left(\chi \right)\sec^{2}\alpha {\left(\tan^{2}\chi -\tan^{2}\alpha \right)}^{-1/2}\sin\chi d\chi ,$$

where *χ* is the angle between **n** and **N**, *f*_D_(*χ*) is the orientational distribution function of the nematic director **n** inside the droplet (i.e., 2*π f*_D_(*χ*)sin *χ*d*χ* gives the fraction of droplet volume where **n** is oriented at an angle between *χ* and *χ* + d*χ* with respect to **N**), and *C* is a proportionality constant. The function *f*_D_(*χ*) is characteristic of the director configuration, and its determination allows calculation of *S*_D_ through Equation (1). The function *G*(*α*) was obtained by deconvolving the experimental profile *I*(*ψ*) (curve A in Figure 25a) with the function *I*_0_(*ψ*) above determined (curve B in Figure 25a), and the result is shown as curve C in Figure 25a. The numerical inversion of the integral Equation (2) made it possible to obtain the droplet director distribution function *f*_D_(*χ*) shown in Figure 25b. Finally, the droplet order parameter was calculated by means of Equation (1), and the result obtained was *S*_D_ = 0.73 ± 0.02 [78]. This value is in excellent agreement both with theoretical calculations of *S*_D_ for bipolar droplets [81,82] and with experimental data of optical phase shift measurements in similar PDLC samples [83].

A similar analysis can be extended to other director configurations and used to discriminate among configurations having the same cylindrical symmetry but differing in the director orientational distribution function, as it occurs for bipolar and axial configurations. As very distinctive patterns correspond to different configurations, this technique represents a unique tool to investigate the dependence of LC ordering and director configuration on the droplet shape and size.

### 4.5. Molecular Director Map

**Figure 26:**
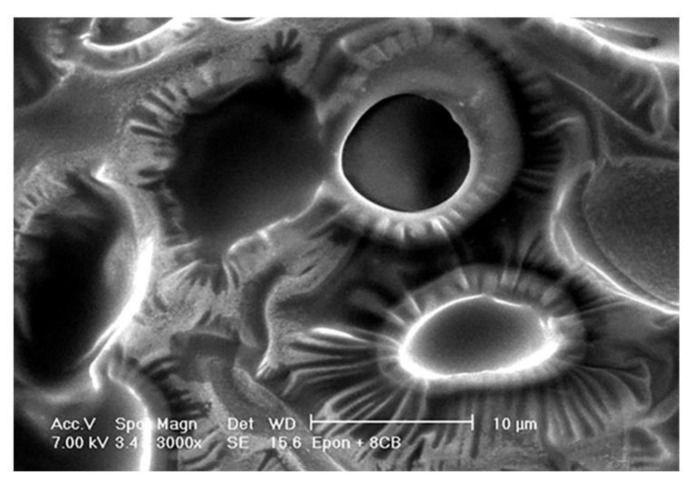
SEM micrograph of geometrically irregular LC dispersions embedded in a polymeric matrix.

More recently, *μ*-XRD has been used to probe the director field configuration in more complex confined geometries, such as those corresponding to PDLCs obtained under unoptimized preparation conditions. In this case, larger and geometrically irregular LC dispersions embedded in the polymeric matrix are obtained, such as those reported as an example in Figure 26.

**Figure 27:**
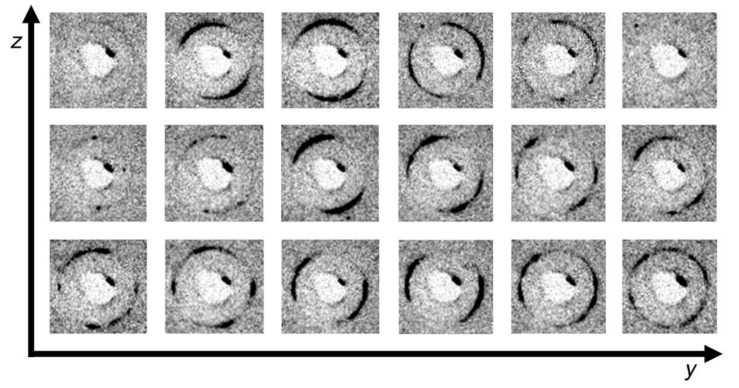
Map of the director field orientation inside the LC volume of an inhomogeneous PDLC film. Horizontal and vertical scans were performed in regular steps of 2 µm.

Here, the complex shape of the confining surface leads to unconventional director configurations that cannot be analytically described. In this case, *μ*-XRD can be used to get an experimental map of the director field orientation inside the LC volume with micrometer resolution. Figure 27 shows an example of the results obtained on a film of the PDLC shown in Figure 26, where the (three) rows and the (six) columns of patterns correspond to horizontal and vertical sample scans, respectively, performed in regular steps of 2 µm.

## 5. Conclusions

We have reviewed a number of XRD experiments performed to study the nanoscale structure of new unconventional LCs and LC-based soft materials with unique physical properties of high fundamental and technological impact. In all cases, synchrotron radiation has proved to be a unique tool to investigate the fine details of the inherent LC order characterizing the relevant mesophase and responsible for such peculiar properties.

The studies performed on BCMs have made great progress in a deeper understanding of the nematic cybotactic order and its key role in determining the polar and biaxial properties of the N phase of these materials. The current challenges to researchers in this field include the following two further steps: (1) the indisputable experimental proof of a spontaneous macroscopically biaxiality in the N phase of BCMs; and (2) the exploration of the extent to which the cybotactic order (and related properties) can be translated to other systems of fundamental and technological interest, e.g., polymeric LCs and more complex ordered fluids, to provide them with tailored unconventional properties.

The results of the XRD studies performed on all-aromatic mesogens such as PPNPP and PPPPP have revealed peculiar features in the XRD pattern of their oriented N phases, offering new insights into the positional short-range order of all-aromatic nematic LCs. The results obtained show that the cybotactic order persists in the N phase of PPPPP as well as PPNPP, thereby suggesting that cybotaxis is a general property of the N phase of all-aromatic mesogens and possibly of all thermotropic LCs. This conclusion has wide-ranging fundamental implications for the current picture of the N phase as a molecular liquid merely characterized by orientational order.

As concerns the PDLCs, the experiments performed have demonstrated the effectiveness of *μ*-XRD as a powerful tool for studying LC ordering and director field configuration in micron-sized confined geometries. In addition, for larger geometries, they have shown how *μ*-XRD offers the unique possibility of probing the spatial dependence on the micrometer length scale of the LC ordering and average director orientation. This technique opens the way to the quantitative study of translational ordering of LCs in the presence of induced disorder as it occurs in highly porous solids or in randomly interconnected networks of pores. Reducing the beam dimension below the micron size, which is now easily accessible, will improve the spatial resolution disclosing the possibility of several new studies, including probing the mesophase/substrate interactions and the influence of segregation on the local order parameter. In addition, it provides a unique tool to study the effects of a strong (sub-micron) confinement on the mesomorphism and phase transition behavior of LCs. Nevertheless, when going down to smaller beam dimensions, radiation damage rather than low flux density becomes the limiting factor of this technique for most organic samples. Single photon counting detectors with a low dark current and low readout noise in connection with further efforts in background reduction will help to push the resolution limits to sub-micrometric length scales. Compared to advanced optical imaging techniques such as multiphoton fluorescence polarization microscopy, which can feature comparable or even better resolution [84,85], *μ*-XRD offers the unique benefit of providing a full structural map of the investigated sample that is not limited to a reconstruction of the molecular director field, but including quantitative information on the main structural parameters characterizing the local orientational and positional order.

Finally, while all the examples discussed in this review refer to nematic LCs, XRD techniques can provide fundamental structural information on many other LC systems, such as lyotropic LCs [86,87,88,89,90,91,92], novel chiral LC phases [93,94,95], LC polymers [96,97], colloidal LCs [98,99,100], etc.
